# Resistance exercise‐induced circulating factors influence the damaged skeletal muscle proteome in a sex‐dependent manner

**DOI:** 10.14814/phy2.70291

**Published:** 2025-04-13

**Authors:** Hui‐Ying Luk, Danielle E. Levitt, Casey R. Appell, Nigel C. Jiwan

**Affiliations:** ^1^ Department of Kinesiology and Sport Management Texas Tech University Lubbock Texas USA

**Keywords:** eccentric exercise, hormones, inflammation, muscle recovery

## Abstract

Muscle recovery after damage is mediated by circulating factors and intracellular signaling pathways. Our previous studies have demonstrated that resistance exercise (RE)‐induced circulating factors elicited sex‐differential responses in damaged muscle. However, the global effects of these circulating factors on damaged muscle are largely understudied. We examined the differential effects of RE‐induced circulating factors and sex on the damaged muscle proteome. Damaged vastus lateralis muscle from 3 men and 3 women from a parent study were analyzed. Participants completed 2 identical bouts of unilateral eccentric knee extensions immediately followed by either upper body RE to induce circulating factors (EXE) or 20‐min seated rest (CON). Muscle biopsies collected from the damaged leg at 24 h were used. 900 proteins were identified by LC–MS/MS analysis. Ingenuity Pathway Analysis was used to detect activation prediction using z‐scores for functional and pathway analyses. In men, 79 proteins were downregulated and 15 were upregulated in EXE versus CON. These differentially expressed proteins were associated with immunological and inflammatory signaling pathways. Biological functions of the differentially expressed proteins in EXE vs. CON in men include inactivating acute inflammatory signaling, neutrophil extracellular trap signaling, ROS production, and activating IL‐12 signaling. These results underline that RE‐induced circulating factors have a sex‐specific effect on the damaged muscle proteome, where immune signaling is altered in men but not women. Given that the immune response is critical for recovery from muscle damage, these results highlight the potential role of RE‐induced circulating factors that could be essential in mediating muscle recovery.

## 1 INTRODUCTION

Recovery from muscle damage requires the orchestration of various physiological processes which can be altered by circulating factors (e.g., cytokines, growth factors, etc.) (Karalaki et al., 2009) and intracellular signaling (e.g., autophagy) (Chen et al., 2022). Some of these circulating factors and cell signals are responsive to exercise and can differ based on sex, age, etc. (Kraemer et al., 1999; Luk et al., 2022; Luk, Appell, et al., 2021; Pierce et al., 2020). Evidence from rodent studies has demonstrated that estrogens are protective against the effects of muscle damage and reduce the subsequent inflammatory response (Tiidus, 2003); however, results from studies comparing men and women are less conclusive (Jomard & Gondin, 2023; Stupka et al., 2000). This could be due to different confounding factors such as inherited muscle fiber type differences, plasma volume shift, training status, etc. (Pizza, 2009). However, when men were supplemented with 17‐β estradiol to isolate the role of this hormone in explaining sex differences in recovery from muscle damage, estradiol reduced neutrophil infiltration and increased antioxidant defenses at 3 and 48 h after exercise‐induced muscle damage (EIMD) (MacNeil et al., 2011). In contrast, findings on the anti‐inflammatory effects of testosterone are mixed (Bianchi, 2019). However, its role in promoting satellite cell myogenic progression is well supported (Velders & Diel, 2013). Similarly, our group has previously demonstrated that resistance exercise (RE)‐induced changes in circulating factors elicit sex‐dimorphic responses in transcription abundance of intramuscular cytokines (Luk, Levitt, et al., 2021) and autophagic markers (Luk, Appell, et al., 2021) as well as protein expression in myogenic markers (Luk et al., 2019) were predominantly observed at 12 h following muscle damage.

The role of RE training in muscle hypertrophy is well elucidated, with evidence showing that the absolute strength and muscle size gains from training can differ by sex; however, relative gains are similar between men and women (Roberts et al., 2020). While it was once hypothesized that transient changes in circulating factors induced by RE might facilitate long‐term adaptations in skeletal muscle growth and strength, particularly in men (Hansen et al., 2001; Rønnestad et al., 2011), more recent research has challenged this notion (Morton et al., 2016; West et al., 2009, 2010; West & Phillips, 2012; Wilkinson et al., 2006). Although these transient hormonal changes do not appear to translate into greater muscle size adaptations from RE training, it remains unclear whether the sex‐specific RE‐induced changes in circulating factors could play a role in the muscle's response to other physiological stimuli, such as muscle damage. We have previously reported lower intramuscular pro‐inflammatory cytokine mRNA levels, greater autophagic flux, and altered markers of mitochondrial dynamics and mitophagy in vastus lateralis (VL) after muscle damage followed by heavy upper body RE than with muscle damage alone (i.e., without heavy RE) in men (Luk et al., 2022; Luk, Appell, et al., 2021; Luk, Levitt, et al., 2021). Because the muscle groups involved in RE were different from those that experienced muscle damage, these differences observed between conditions in men were attributable to circulating factors. However, these physiological processes that are critical to muscle recovery were not different between conditions in women. Despite the presence of sex‐specific responses in these selected physiological processes based on preexisting knowledge of the role of sex hormones, other underlying physiological and molecular responses in damaged skeletal muscle between men and women remain largely unknown.

Much of the work aiming to understand the role of circulating factors in skeletal muscle adaptation has focused on hormones, with emphasis on male sex hormones. Such effects have been studied using androgen administration at supraphysiological dosages (Sinha‐Hikim et al., 2003, 2006) and androgen receptor knockout and transfection models (Sakakibara et al., 2021; Yin et al., 2020). Our knowledge on RE‐induced modulation of endogenous hormones and other circulating factors on muscle, particularly damaged muscle, is largely underinvestigated. This study is particularly noteworthy because the repair of muscle damage requires coordinated responses from immune, inflammatory, myogenic, and anabolic/catabolic signaling pathways for effective repair and adaptation. Individuals at all ages engaging in recreational exercise routinely encounter exercise‐induced muscle damage (EIMD) notably during activities that include a substantial eccentric component (e.g., downhill running, squats—particularly when executed with increased load or volume stiff‐legged deadlifts, and rapid decelerations in sports). Similar instances of muscle damage may occur following falls, unaccustomed stair descents, and comparable events. Given the critical importance of proper recovery for muscle health, it is imperative to investigate the role of circulating factors in the molecular processes pivotal to muscle regeneration.

While the majority of studies on human muscle gene sequencing and proteomic profiling have focused on RE and endurance training in men, recent research has begun to elucidate sex differences in the physiological and molecular characteristics of muscle in both untrained and endurance‐trained individuals (Emanuelsson et al., 2024). However, sex differences in the physiological and molecular profiles of skeletal muscle during recovery from exercise remain largely unexplored (Deane et al., 2022; Hesketh et al., 2020). Additionally, our previous findings revealed sex‐specific transcriptional responses for markers crucial to muscle repair at 12 h following muscle damaging RE; these transcriptional differences were driven by differences in RE‐induced circulating factors (Peake et al., 2017). The early phase of recovery (4–24 h) after muscle damage is critical to muscle regeneration and may impact muscle adaptation (Peake et al., 2017). Given our previous findings and the importance of the early recovery period to muscle adaptation, implementing an unbiased, global approach to comprehensively examine sex differences in the skeletal muscle proteome during the early exercise recovery period is warranted. This approach offers a unique opportunity to capture both known and previously uncharacterized molecular pathways involved in muscle recovery, contributing to a deeper understanding of the complex physiological responses to muscle damage across sexes.

## 2 METHODS

This study utilized a within‐subjects, randomized, counterbalanced crossover design. The details of the study design, RE protocol, and sample collection timelines have been previously published (Luk et al., 2019). Briefly, a total of 19 untrained young adult participants completed the study. For this report, muscle samples from 3 men and 3 women were analyzed (Table 1) and were selected based on sample availability. Participants provided informed consent, and this study was approved by the University of North Texas Institutional Review Board (#15–351) and adhered to the Declaration of Helsinki. Women who participated in the study had regular menstrual cycles, and all experimental visits were completed during the early follicular phase when estrogen concentration is at its lowest and does not increase with RE (Nakamura et al., 2011).

**TABLE 1:** Demographic characteristics of study participants. Values are presented as means ± SD.

	Men (*n* = 3)	Women (*n* = 3)
Age (yr)	23 ± 5	19 ± 1
Height (cm)	181.6 ± 5.9	162.5 ± 16.4
Weight (kg)	86.0 ± 15.8	53.5 ± 6.0
Lean mass (kg)	61.2 ± 5.1	33.9 ± 4.2
Fat (%)	24.9 ± 9.7	32.6 ± 12.9

## 3 EXPERIMENTAL VISITS

### 3.1 Exercise protocol

To examine the role of RE‐induced circulating factors on changes in the skeletal muscle proteome following exercise‐induced damage, participants completed two identical sessions of unilateral eccentric knee extensions to induce muscle damage (8 sets of 10 maximal‐effort repetitions with 3 min of rest between sets). The detailed study protocol has been published previously (Luk et al., 2019). Our published data show that this protocol increased circulating creatine kinase concentrations and subjective VAS pain scale 12 and 24 h later (Luk, Levitt, et al., 2021), indicating that muscle damage was effectively induced. Participants then immediately proceeded to complete heavy upper body RE (EXE condition) or seated rest (20 min, time‐matched; CON condition). The upper body RE protocol consisted of 4 sets of 10 repetitions of bench press, bench row, and seated overhead press exercises at 80% of 1 repetition maximum with 1 min rest between sets and exercises. The upper body RE induced an acute increase in canonical anabolic and stress hormones, whereas this response was absent in the CON condition (Luk et al., 2019). Since the magnitude of lower body muscle damage was similar in both conditions, and the leg muscles were not further exercised in the EXE condition, this design allows for examination of the role of RE‐induced circulating factors on damaged muscle.

### 3.2 Sample collection

The detailed sample collection and processing procedures have been published previously (Luk et al., 2019). Briefly, participants arrived at the laboratory in the morning after an overnight fast. Hydration status was assessed, and a baseline (BL) blood sample was collected. Muscle samples were collected at the BL, 12 h, and 24 h time points and immediately flash‐frozen in liquid nitrogen.

Our previous data showed significant differences in the transcriptional abundance of key pro‐inflammatory (CCL2, TNF‐alpha, IL‐6), anti‐inflammatory (IL‐10) (Luk, Levitt, et al., 2021), and markers of mitophagy and mitochondrial fission (MFN1, PINK1, DRP1) (Luk et al., 2022) between men in EXE and CON conditions, as well as between men and women in EXE at 12 h following exercise‐induced muscle damage. For this report, we performed proteomic analyses using muscle samples collected 24 h after exercise. The 24 h time point was selected as it captures the critical window where both transcriptional responses and their translation into functional proteins occur.

## 4 DISCOVERY‐BASED PROTEOMICS

### 4.1 Sample preparation

Muscle samples (30 mg) were washed with 50 mM ammonium bicarbonate (ABC) buffer, and extraction buffer (50 mM ABC buffer, 5% sodium deoxycholate (SDC)) was added. The samples were homogenized by zirconium beads using a Beadbug microtube homogenizer (Benchmark Scientific, Edison, NJ). The homogenization was performed at 4°C and 4000 rpm for 30 s, followed by a 30 s pause. This step was repeated 6 times. After centrifuging for 15 min, the supernatant containing extracted proteins was collected and diluted 1:10 in 50 mM ABC buffer. Protein concentration was determined using a bicinchoninic acid (BCA) assay (ThermoFisher Scientific, Rockford, IL) following the manufacturer's instructions.

ABC‐SDC solution was added to 20‐μg aliquots of each extracted protein sample to a volume of 50 μL. Samples were then subjected to reduction, alkylation, and tryptic digestion. Proteins were thermally denatured at 80°C for 10 min. Reduction was performed by adding a 1.25 μL of 200 mM dithiothreitol (DTT) and incubating at 60°C for 45 min. Reduced proteins were then alkylated by adding 5 μL of iodoacetamide (IAA, 200 mM) and incubated at 37°C in the dark for 45 minutes. To quench excess IAA, another 1.25 μL of DTT was added, and samples were incubated at 37°C for 30 min. Following reduction and alkylation, trypsin (Promega, Madison, WI) was added at a ratio of 1:25 (enzyme: protein, w/w) into samples and incubated at 37°C for 18 h. After incubation, formic acid was added at a final concentration of 0.5% (v/v) for the dual purposes of quenching the enzymatic reaction and removing the SDC detergent. Samples were then mixed thoroughly and centrifuged at 21,100 ***g*** for 10 min. The supernatant was collected, speed‐vac dried, and resuspended in aqueous solution containing 2% acetonitrile (ACN) and 0.1% formic acid (FA) prior to LC–MS/MS analysis.

### 4.2 LC–MS/MS measurement

Aliquots of 1 μg tryptic digests were subjected to the untargeted proteomic analysis. A Dionex 3000 Ultimate nano‐LC (Dionex, Sunnyvale, CA) interfaced with a QExactive HF mass spectrometer (Thermo Scientific, San Jose, CA) equipped with an EASY‐Spray source was used for the analysis. Tryptic digests were first loaded to an Acclaim PepMap100 C18 guard column (3 μm, 100 Å, Dionex) at a flow rate of 3 μL/min for on‐line desalting. Next, the peptide separation was achieved using an Acclaim PepMap100 C18 capillary column (75 μm id × 150 mm, 2 μm, 100 Å, Dionex) at 0.35 μL/min over 120 min. The mobile phase A contained 2% ACN, 0.1% FA, and 97.9% water, while mobile phase B contained 0.1% FA in ACN. The LC gradient was as follows: solvent B was kept at 5% for the first 10 min, increased from 5% to 20% over 55 min, 20% to 30% over 25 min, 30% to 50% over 20 min, 50% to 80% over 1 min, kept at 80% for 4 min, decreased from 80% to 5% over 1 min, and finally maintained at 5% over 4 min.

The QExactive HF was used in data dependent acquisition mode. The scan events were set as a full MS scan of m/z 400–1600 at a mass resolution of 60,000, followed by an HCD MS/MS scan repeated on the 20 most intense ions selected from the previous full MS scan with an isolation window of m/z 1.5. The normalized collision energy was set to 25%, and the dynamic exclusion was set at 30 s.

### 4.3 LC–MS/MS data analysis

The raw data obtained from LC–MS/MS analysis were processed with the MaxQuant software version 1.6.3.4. A database search was performed against the UniProtKB/Swiss‐Prot Homo sapiens database. The search included cysteine carbamidomethylation as a fixed modification and variable modifications, including methionine oxidation and acetylation of the protein N‐terminal. Trypsin was specified as the proteolysis enzyme, and a maximum of 2 missed cleavages were allowed.

For identification, the peptide precursor mass tolerance was 20 pm in the first search and 4.5 pm in the main search. A deviation of 0.5 Da was allowed for fragment matching. Only peptides with a minimum length of 7 amino acids were considered for identification. The false discovery rate (FDR) was set at 0.01 at both peptide and protein levels, which controls the proportion of false positives when identifying peptides and proteins, ensuring stringent criteria for identification. The minimum ratio count was set at two to determine the intensities of proteins for label‐free quantification (LFQ). Both unique and razor peptides were considered for quantification.

Data were processed using Perseus software version 1.6.2.3. Protein IDs were filtered to eliminate identifications from the reverse database, and proteins were only identified by site and common contaminants. Student's *t*‐test analyses were carried out using Perseus software, and only the proteins with a *p* value <0.05 were considered to be differentially expressed between groups (Tables S1–S4).

### 4.4 Principal component analyses

Abundance ratios were calculated by pairwise comparisons of condition (EXE vs. CON) for each sex and sex (men vs. women) for each condition. Bioinformatic analyses were performed using Qiagen Ingenuity Pathway Analysis (IPA) software (QIAGEN, Inc.). To be considered in the core analysis for identifying enriched biological processes and functions, proteins had at least 1.0‐fold differential expression, and a *p* value <0.05 was set for IPA. Principal component analysis (PCA) was performed on the proteomic data using scikit‐learn version 1.0.2 Python code library. In addition, z‐scores were calculated using IPA to predict changes in associated biological functions. IPA was not conducted for comparisons where the number of differentially expressed proteins was low.

## 5 RESULTS

### 5.1 Protein identification and quantification – Effects of condition within sex

**FIGURE 1:**
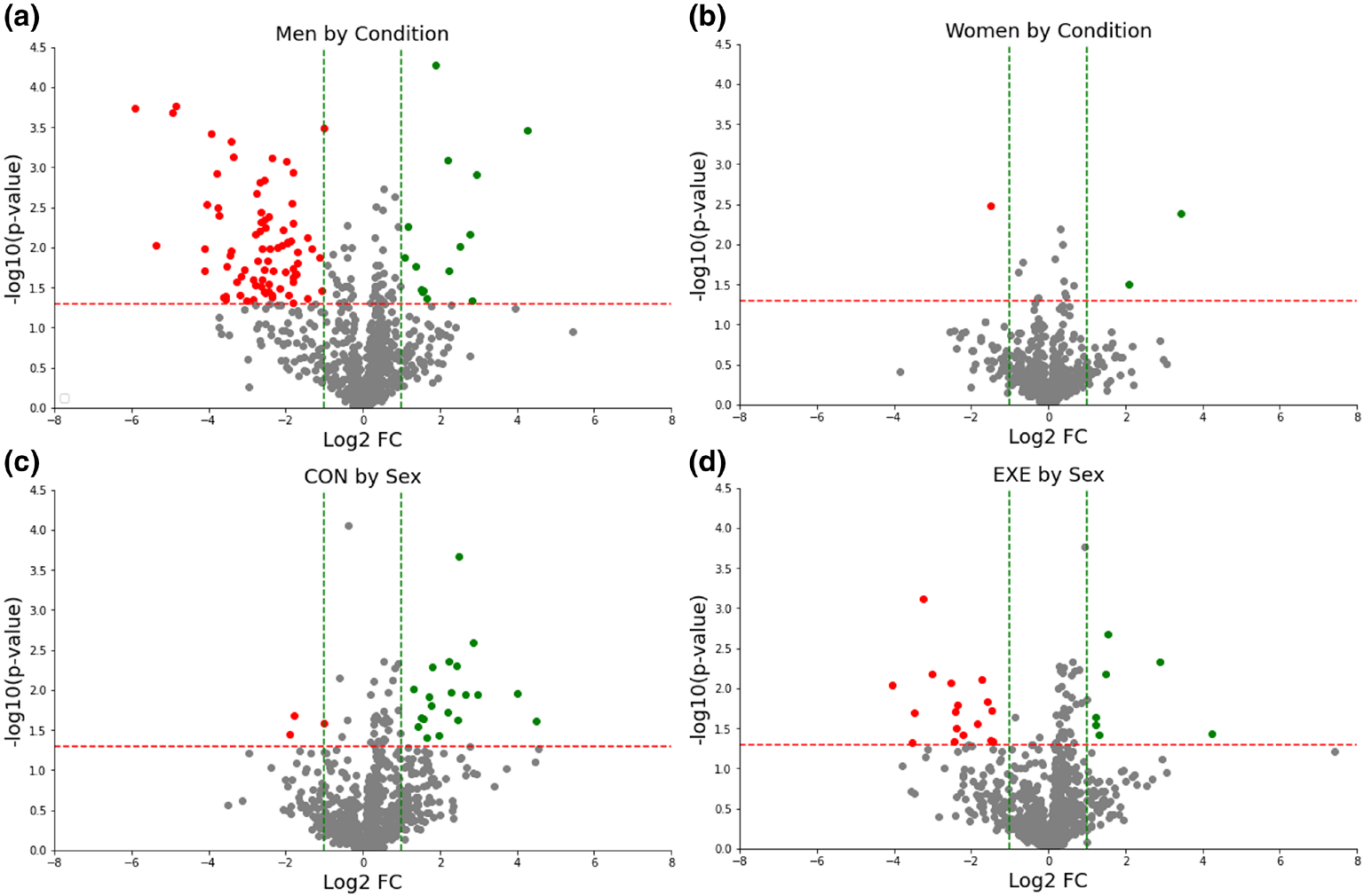
Graphical representation of the damaged muscle tissue proteomics data based on condition (EXE vs. CON) in men and women using volcano plots. Plots depict condition effects (EXE vs. CON) in men (a) and women (b) and sex effects (men vs. women) in CON (c) and EXE (d) at 24 h after the exercise protocol shown as *p‐*value and fold change. Red points in the volcano plot represent data points with *p* ≤ 0.05 and a fold change of ≤−1.0. Green points in the volcano plot represent data points with *p* ≤ 0.05 and a fold change of ≥1.0.

A total of 900 expressed proteins were identified for each sample. In men, there were 79 significantly downregulated and 15 upregulated proteins in EXE compared to CON at 24 h after muscle damage (Figure 1a). In women, there was 1 significantly downregulated and 2 upregulated proteins in EXE compared to CON at 24 h after muscle damage (Figure 1b).

### 5.2 Protein identification and quantification – Effects of sex within condition

In CON, there were 17 significantly downregulated and 7 upregulated proteins in men compared to women at 24 h after muscle damage (Figure 1c). In EXE, there were 3 significantly downregulated and 20 upregulated proteins in men compared to women at 24 h after muscle damage (Figure 1d).

### 5.3 Principal component analysis

**FIGURE 2:**
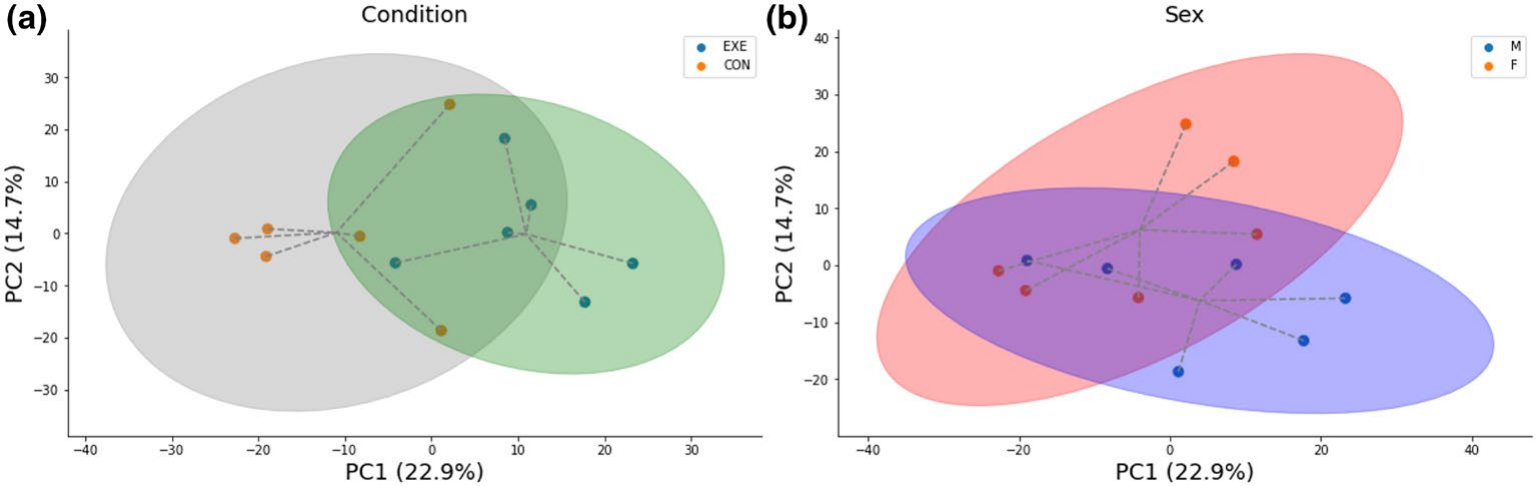
Principal component analysis (PCA) plots of the proteomics data. Plots depict that the global proteomic differences by (a) condition (EXE vs. CON) separate most substantially along principal component (PC) 1 which accounts for 22.9% of variability in the data, and (b) sex (men vs. women) separate most substantially along PC 2, which accounts for 14.9% of variability in the data. Cluster ellipses are 95% confidence intervals around the estimated cluster's mean.

PCA was utilized to compare the proteomic profiles from all groups. Principal component (PC)1 accounted for 22.9% of the variance, PC2 accounted for 14.7% of the variance, and PC3 accounted for 11.9% of the variance in the data (Figure S1A–D). For PC1, data appear to cluster by condition (Figure 2a), whereas for PC2, data appear to cluster by sex (Figure 2b).

### 5.4 Canonical pathways by sex

**FIGURE 3:**
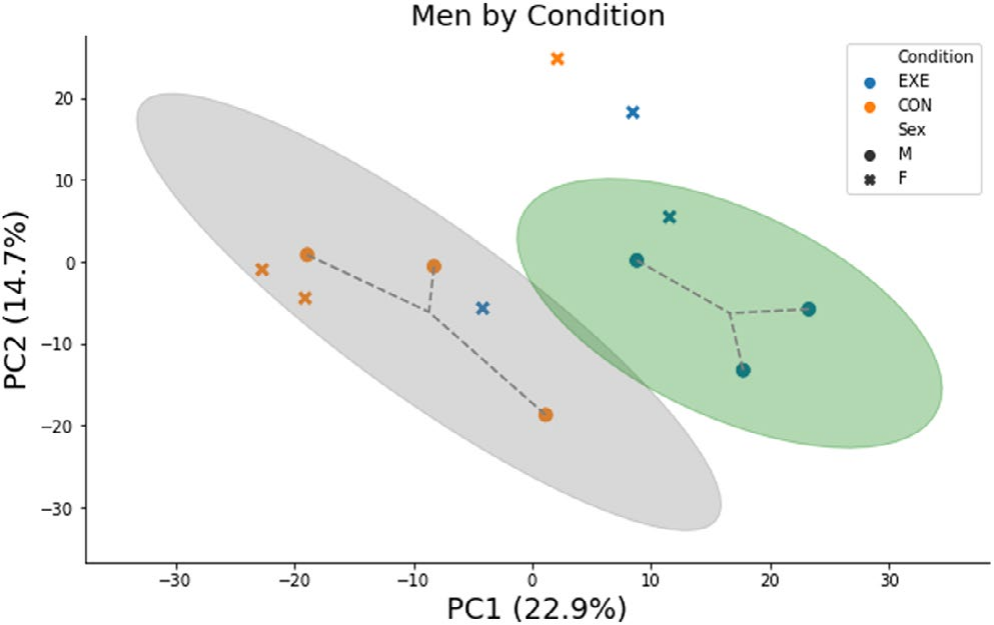
Proteomics profiles of condition (EXE vs. CON) for men separated along PC1. Cluster ellipses are 95% confidence intervals around the estimated cluster's mean.

**FIGURE 4:**
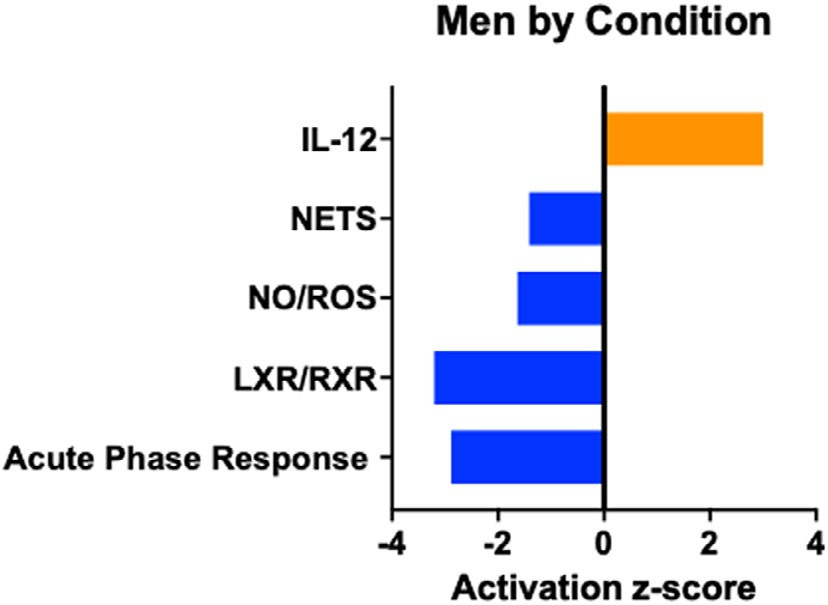
Canonical pathways associated with protein changes between EXE and CON in men with calculated z‐scores. Negative z‐score indicates pathway inhibition, and positive z‐score indicates pathway activation.

In men, across conditions, the top enriched canonical pathways included the “acute phase response signaling”, “liver X receptor/retinoid X receptor (LXR/RXR), ‘farnesoid X receptor (FXR)/RXR’, ‘interleukin (IL)‐12 signaling and production in macrophages’, and ‘production of nitric oxide (NO) and reactive oxygen species (ROS) in macrophages’”. With the enriched pathways, an additional PCA analysis was completed for men across condition and the separation between EXE and CON was along PC1 (Figure 3). The z‐scores were calculated and used as predictors of directionality for each pathway. Significant directionality was observed, and z‐scores indicated decreased activation of acute phase response signaling, LXR/RXR signaling, production of NO and ROS in macrophages, and neutrophil extracellular trap (NET) signaling pathways and increased activation of the macrophage IL‐12 signaling and production pathway in EXE versus CON (Figure 4).

## 6 DISCUSSION

Using a quantitative, discovery‐based approach, we assessed differences in protein profiles due to RE‐induced circulating factors in the vastus lateralis after exercise‐induced muscle damage in young, untrained men and women. Functional pathway analyses of these differentially expressed proteins predominantly pointed toward the immune profile. Specifically, predictions based on this immune pathway analysis indicated inhibition of acute phase response, NET signaling, and the production of NO and ROS in macrophages due to RE‐induced circulating factors in men. Moreover, there was activation of IL‐12 production in macrophages when upper body RE was performed following muscle damage. Together, these findings showed that after 24 h of recovery from muscle damage, RE‐induced circulating factors mediated changes in cellular immune response signaling in damaged muscle for men, but not women.

After tissue damage, the immediate acute phase response arises from the infiltration of inflammatory cells, first clearing damaged tissue, then promoting regeneration and restoring homeostasis (Serrano et al., 2018). Specifically, Fielding et al. proposed that the mechanical stress from eccentric exercise results in initial damage particularly at the Z‐disks. Subsequently, it creates a chemotactic gradient to attract neutrophils for initiating the inflammatory response (Fielding et al., 1993). This inflammatory response includes phagocytosis by neutrophils, and one mechanism of neutrophil action is NET formation (Rosales, 2018). While NET formation is commonly linked to disease‐related inflammatory conditions (Ma et al., 2023; Mutua & Gershwin, 2021), some evidence suggests that intense exercise can increase circulating NETs in humans (Beiter et al., 2014; Orysiak et al., 2021; Syu et al., 2013). However, the role of net in exercise‐induced muscle damage and regeneration remains largely unexplored. In other models of injured muscle, reducing NET formation has been associated with decreased fibrosis, suggesting a potential influence of NET formation for muscle regeneration (Edwards et al., 2020). While the exact physiological functions, triggers, and formation pathways of NETs are not fully understood, they are commonly associated with damage‐associated molecular patterns (DAMPs), pathogen defense, and inflammatory cytokine response (Boeltz et al., 2019). In hepatocytes, phorbol‐12‐myristate 13‐acetate and superoxide have been shown to enhance NET formation, which can be suppressed by NADPH oxidase (NOX) inhibitors and toll‐like receptor (TLR)4 knockout (Al‐Khafaji et al., 2016). DAMPs, known stimulators of TLR4, have been implicated in nitric oxide (NO) and ROS production in macrophages (Park et al., 2021), as well as in driving NET formation (Ma et al., 2023) and inflammatory cytokine production. Given that the current eccentric knee extension protocol has been shown to induce muscle damage (Luk, Levitt, et al., 2021), it is possible that DAMPs (e.g., high mobility group box 1, etc.) could contribute to a TLR‐4 mediated inflammatory response and influence NET formation. Additionally, in ischemia–reperfusion injury, reduced NET formation in skeletal muscle was linked to TLR 7/8/9 inhibition and decreased systemic concentrations of IL‐10 and TNF‐α (Edwards et al., 2020). Our IPA analysis, in conjunction with previous findings demonstrating lower *IL10* and *TNFA* transcription (driven by men) in damaged muscle in response to RE‐induced circulating factors, aligns with earlier observations by Edwards et al. (2020). Further research is needed to clarify the physiological roles of NETs in skeletal muscle recovery from EIMD and to determine whether RE‐induced circulating factors modulate NET formation.

One potential mechanism that could, at least in part, explain these findings is the influence of sex hormones. Among the most well‐characterized circulating factors induced by RE are sex and stress hormones. Notably, intense RE elicits an acute, transient increase in testosterone concentrations in men but not in women. Neutrophils express androgen receptors, and elevated androgen levels can increase neutrophil maturation and function (Markman et al., 2020), which in turn may promote NET formation. Interestingly, men with COVID‐19 who exhibited reduced circulating testosterone had higher LL‐37, an antimicrobial peptide that stabilized NETs and protected them from degradation (De Buhr et al., 2022). These data might suggest that although testosterone may facilitate NET formation through neutrophil activation, it could also promote NET degradation. Testosterone and estrogen are known to have anti‐inflammatory properties (Barbosa et al., 2021; Bubak et al., 2024; Malkin et al., 2004). Although speculative, here, the transient increase in RE‐induced testosterone in men might exert sufficient anti‐inflammatory effects on the injured muscle. Conversely, in women, the absence of differences in the immune functional pathways between conditions is aligned with our prior results, where no changes were detected in intramuscular cytokine transcripts (Luk, Levitt, et al., 2021). This lack of responses could be partly explained by estrogen's protective role in maintaining muscle cell membrane integrity; therefore, the extent of muscle damage induced by the damaging protocol and subsequent inflammatory response (Tiidus, 2003) may have been limited regardless of condition. Additionally, the lack of differences noted between men and women might have been influenced by rigorous statistical approaches that made differences too small to detect and the protective effects of both testosterone and estrogen. Notably, Shi et al. reported that aromatase activity, which converts testosterone to estrogen, increases in skeletal muscle, accompanied by an increase in the intramuscular estrogen concentration following exercise in ovariectomized rats (Shi et al., 2019). While speculative, this suggests that testosterone in men may provide some protective effects through local conversion to estrogen in skeletal muscle. Further research is needed to clarify the interplay between sex hormones, NET formation, and inflammatory responses to exercise‐induced muscle damage following EIMD.

In addition, proteomic analyses revealed decreased expression of proteins in the NO and ROS production pathways, alongside increased expression of proteins in the macrophage IL‐12 production pathway in men when RE followed muscle damage compared to rest. These pathways are closely linked to transforming growth factor (TGF)‐β signaling, which can both regulate and be regulated by TGF‐β. Notably, our previous findings showed increased *TGFB* gene expression in men following RE after muscle damage (Luk, Levitt, et al., 2021). TGF‐β is known to suppress interferon (IFN)‐γ production (Vodovotz et al., 1993). Since IFN‐γ plays a critical role in macrophage activation and stimulates NO production (Blanchette et al., 2003), its inhibition by TGF‐β could lead to reduced NO production (Vodovotz et al., 1993), aligning with our current findings. Consequently, the reduction in NO might promote macrophage IL‐12 production (Xiong et al., 2004). Further investigation is needed to determine the implications of these pathway responses, particularly whether sex differences influence the dynamics of NO and IL‐12 in regulating TGF‐β signaling and macrophage activation during muscle regeneration.

The design of this study represents one of its key strengths. This is the first study to distinguish the effects of resistance exercise (RE)‐induced circulating factors from those of continued localized metabolic and mechanical stressors on muscle during the early post‐EIMD period in men and women. This allowed us to explore the role of circulating factors resulting from RE on enrichment of functional proteomic pathways within damaged skeletal muscle. However, this report is not without limitations. For example, we were only able to capture the proteome at 24 h post‐EIMD. Muscle recovery processes extend much longer (96 h to 30 days depending on the extent of damage) (Järvinen et al., 2005). However, early recovery processes are critical to the coordinated repair process (Tidball, 2017), highlighting the importance of the 24‐h timepoint. In addition, the use of a small number of samples for analysis could limit the scope of the interpretation of the global proteomic analysis. We acknowledge that the use of a small number of samples for analysis could limit the scope of the interpretation in the global proteomic analysis. However, small sample sizes are common in proteomic analyses because of the importance of including all samples in the same run (Deshmukh et al., 2021; Hoffman et al., 2015; Kurgan et al., 2019; Potts et al., 2017). Moreover, prior proteomics‐based investigations in exercise science have utilized small sample sizes (e.g., 4 healthy young adult men, 6 mice [3 mice/group]), which were adequate for detecting differences in proteomic profiles following exercise (Deshmukh et al., 2021; Hoffman et al., 2015; Kurgan et al., 2019; Potts et al., 2017). Notably, the cytokines transcriptional data from a previously published report (Luk, Levitt, et al., 2021) align with the findings in the present study. However, it is noteworthy to mention that post‐transcriptional and post‐translational regulatory mechanisms contribute to the complexity of interpreting these results. Together, these data suggest that a sex differential response in exercise‐induced circulating factors could potentially influence the pro‐inflammatory state in damaged muscle at 24 h following exercise‐induced damage. It is noteworthy to mention that despite lower *TGFB*, *IL10*, *TNFA* mRNA for women compared to men previously observed at 24 h regardless of conditions, those previous findings were targeted cytokine gene expression measurements. Our current report focuses on protein enrichment to examine signaling pathways in their entirety.

Malm and Yu argued that voluntary eccentric exercise primarily induces muscle remodeling, particularly in the Z‐band region, rather than causing significant muscle necrosis or inflammation (Malm & Yu, 2012). However, this study conducted a proteomic analysis on muscle samples collected 48 hours after a 45‐min downhill run at a −8% incline at 60% of VO_2max_. Prior research has extensively documented the pro‐ and anti‐inflammatory immune responses following muscle damage, and the inflammatory responses to muscle damage heighten within the initial 24 h and typically while neutrophil infiltration subsides by 48 h, but macrophage infiltration does not (Peake et al., 2017). Rodent studies further highlight the importance of the inflammatory response, as the depletion of immune cells has been shown to impair muscle regeneration (Arnold et al., 2007). This underscores the critical role of the immune system in clearing damaged debris and facilitating muscle repair, which is necessary for subsequent muscle adaptation. The concept of muscle adaptation to damage is illustrated by the repeated bout effect, which refers to the reduction in muscle damage during repeated exposure to the same muscle‐damaging exercise (Ye et al., 2021). This concept has been illustrated by Evans et al., who demonstrated that endurance trained individuals experience less muscle damage than untrained individuals due to the adaptive response from training (Evans et al., 1986). In the present study, however, the two identical muscle‐damaging exercises were performed on different legs, thus preventing the repeated bout effect from influencing the outcomes.

A limitation of this study is the relatively small sample size, which may impact the generalizability of the findings. However, given the repeated‐measures design, each participant served as their own control, thereby strengthening internal validity and reducing inter‐individual variability. Due to the limited sample size, we did not perform factorial statistical analysis, as it would require greater power to reliably detect interactions. Instead, we conducted four independent *t*‐tests without multiple comparison correction (e.g., FDR correction), which increases the risk of type I error. However, given the small sample size and the exploratory nature of this report, applying FDR correction could be overly conservative and potentially eliminate biologically meaningful observations. Although this study utilized a within‐subject crossover design, we performed independent *t*‐test analyses when comparing conditions within each sex to allow for a more comprehensive comparison while accounting for missing protein values. Notably, the observed differences, particularly the differential protein expression in immunological and inflammatory pathways in men between conditions and the lack of significant changes in women, align with our previous findings (Luk, Levitt, et al., 2021), supporting the biological relevance of these results. However, future studies with larger sample sizes and appropriately powered analyses will be necessary to confirm these results and further explore potential sex‐specific responses in damaged muscle.

## 7 CONCLUSION

While the majority of studies on human muscle gene sequencing and proteomic profiling have focused on RE and endurance exercise training, the proteomic responses to muscle damage remain poorly understood (Sakakibara et al., 2021; Sinha‐Hikim et al., 2006). This study is the first to examine the impact of RE‐induced circulating factors on the skeletal muscle proteomic profile during the early phase of recovery from EIMD in men and women. We demonstrated that these circulating factors altered functional proteomic pathways involved in immune responses in injured skeletal muscle of men only, representing a critical sex‐specific effect that is relevant for individuals who may experience muscle damage due to exercise or other causes. That is, the results suggest that men who experience substantial muscle damage may consider performing RE using non‐injured muscle to attenuate the magnitude of the intramuscular inflammatory response. However, additional studies to determine the downstream impacts on muscle repair and recovery are warranted to further substantiate this recommendation. Other critical proteomic pathways enriched in the present study did not differ by sex or condition, suggesting that these sex‐specific and circulating factor‐driven effects are not global. Given the importance of the immune response in healing damaged muscle tissue, these results highlight the potential importance of less understood mediators in shaping our understanding of sex‐mediated exercise‐induced circulating factors in muscle repair.

## AUTHOR CONTRIBUTIONS

H.Y.L. conceived and designed the research; H.Y.L. and D.E.L. performed the experiment; H.Y.L. analyzed the data; H.Y.L. and D.E.L. interpreted the results of the experiments; H.Y.L. prepared the figures; H.Y.L. drafted the manuscript; D.E.L., C.R.A., and N.C.J. revised the manuscript; all authors approved the final version of the manuscript.

## FUNDING INFORMATION

The research was supported in part by a grant from the National Strength and Conditioning Association Foundation and Texas Tech University Internal Funding.

## CONFLICT OF INTEREST STATEMENT

No conflicts of interest, financial or otherwise, are declared by the authors.

## ETHICS STATEMENT

Participants provided written informed consent to participate. The study was reviewed and approved by the University of North Texas Institutional Review Board (#15‐351).

## DISCLAIMERS

The content is solely the authors' responsibility and does not necessarily represent the official views of Texas Tech University.

## Supporting information


Figure S1.

Table S1.
