# Supplementary figures and images for: Resistance exercise‐induced circulating factors influence the damaged skeletal muscle proteome in a sex‐dependent manner
Source: Physiol Rep. 2025 Apr 13;13(7):e70291. doi: 10.14814/phy2.70291 (PMC11994862; doi:10.14814/phy2.70291)

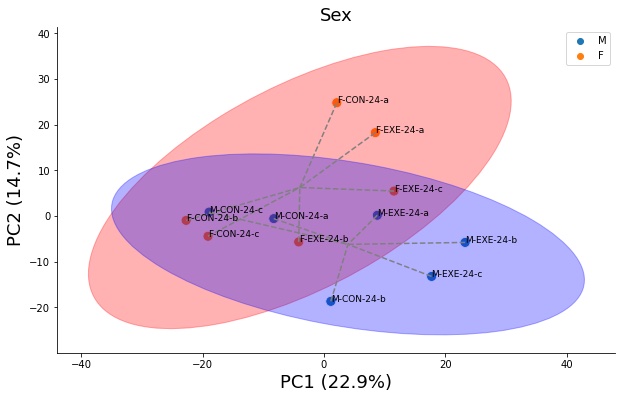

Supplement: Supplementary file 1 — Figure S1. [file PHY2-13-e70291-s001.zip › PHYSREP-2024-08-561-f05-z-_FigS1A.jpg]

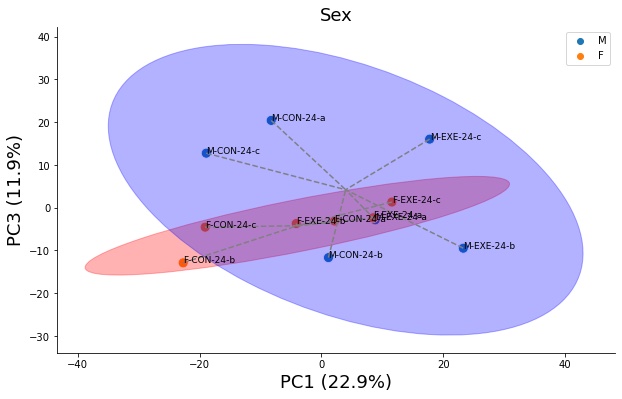

Supplement: Supplementary file 1 — Figure S1. [file PHY2-13-e70291-s001.zip › PHYSREP-2024-08-561-f06-z-_FigS1B.jpg]

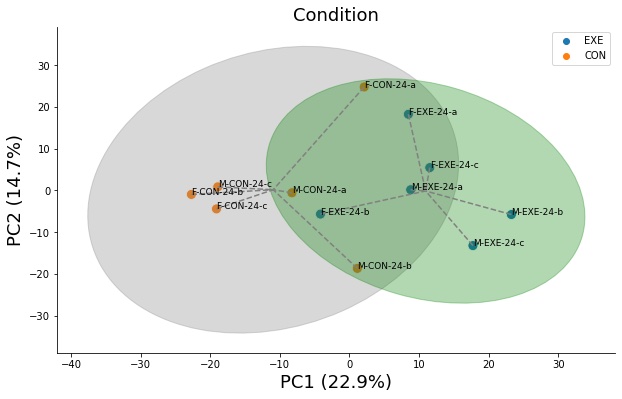

Supplement: Supplementary file 1 — Figure S1. [file PHY2-13-e70291-s001.zip › PHYSREP-2024-08-561-f07-z-_FigS1C.jpg]

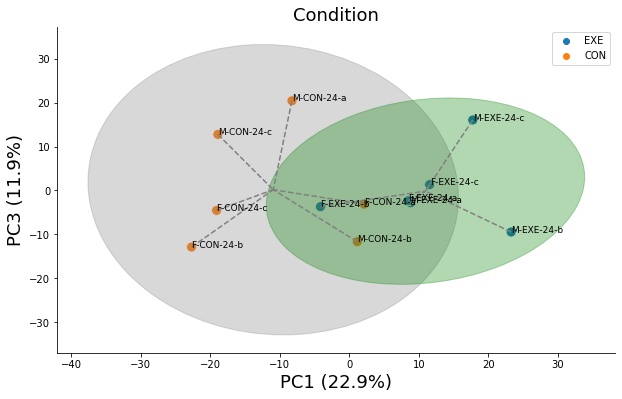

Supplement: Supplementary file 1 — Figure S1. [file PHY2-13-e70291-s001.zip › PHYSREP-2024-08-561-f08-z-_FigS1D.jpg]
